# DL-Selenomethionine Alleviates Oxidative Stress Induced by Zearalenone via Nrf2/Keap1 Signaling Pathway in IPEC-J2 Cells

**DOI:** 10.3390/toxins13080557

**Published:** 2021-08-10

**Authors:** Haoyang Sun, Meiling Zhang, Jianping Li, Anshan Shan

**Affiliations:** Institute of Animal Nutrition, College of Animal Science and Technology, Northeast Agricultural University, Harbin 150030, China; sunhaoyang@neau.edu.cn (H.S.); zhangmeiling202020@163.com (M.Z.)

**Keywords:** zearalenone, selenomethionine, IPEC-J2, oxidative stress, Nrf2/Keap1 signaling

## Abstract

Zearalenone (ZEN) is a kind of nonsteroidal mycotoxin that is considered a risk affecting the safety of human food and livestock feed that causes oxidative damages in mammalian cells. Selenomethionine (SeMet) was indicated to have antioxidant activity and received great interest in investigating the role of SeMet as a therapeutic agent in oxidation. Therefore, the aim of this study was to investigate the hormetic role of DL-SeMet in porcine intestinal epithelial J2 (IPEC-J2) cells against ZEN-induced oxidative stress injury. As a result of this experiment, 30 μg/mL of ZEN was observed with significantly statistical effects in cell viability. Following the dose-dependent manner, 20 μg/mL was chosen for the subsequent experiments. Then, further results in the current study showed that the ZENinduced oxidative stress with subsequent suppression of the expression of antioxidant stress pathway-related genes species. Moreover, SeMet reversed the oxidative damage and cell death of ZEN toxins to some extent, by a Nrf2/Keap1-ARE pathway. The finding of this experiment provided a foundation for further research on the ZEN-caused cell oxidative damage and the cure technology.

## 1. Introduction

Zearalenone (ZEN, as well as ZEA), is a kind of nonsteroidal mycotoxin produced as the secondary metabolite by many species of Fusarium fungi such as *F. graminearum*, *F. culmorum*, and *F. verticillioides* [1]. It accumulates in crops under poor storage conditions, not only preharvest but also postharvest [2]. It is a widespread food contaminant that is commonly detected in cash crops, including maize, wheat, barley, sorghum, rye, and other grains [3]. Its occurrence in other human daily food products such as soybean products, dried fruit and vegetables, and milk products has also been reported [4,5]. Among different livestock, swine proved to be highly sensitive to the effects of ZEN mycotoxin via contaminated feed. Maize is the major energy feed in the swine diet and is very prone to Fusarium mycotoxin contamination. In addition, a genetic similarity exists between humans and swine, which makes a suitable model for the study of various deleterious effects caused by ZEN.

Research into ZEN has intensified for many years and a number of studies have reported various deleterious effects caused by ZEN, but many of the observed damages are, presumably, not always caused by interfering in the physiological estrogen signaling pathways [6]. Recently, several studies showed that ZEN is cytotoxic through inhibition of cell viability and apoptosis and induces the stress response in different cultured cell lines [7,8]. Oxidative damage is the predominant cause of cell injury and cell death. It has become increasingly clear that ZEN can produce reactive oxygen species (ROS) in mammalian cells [9]. Among multiple mechanisms, the Nrf2-Keap1 signaling pathway is a key transcription factor in the regulation of antioxidant protection [10]. Nrf2 has the ability to control many different aspects of cellular protection.

Selenomethionine (SeMet) is the dominant form of organic selenium compounds derived from the diet. SeMet has 3-times-higher incorporation into RBS compared with selenite [11]. These compounds are efficiently absorbed in the intestine and show a high bioavailability and generally lower toxicity [12,13]. In addition, selenium could protect the liver against arsenic-induced toxicity through activation of the Nrf2 pathway [14]. Thus, it was hypothesized that SeMet may promote the survival of IPEC-J2 cells against oxidative stress via activating the Nrf2/Keap1-ARE signal pathway.

In the present study, we determined the role of DL-SeMet on IPEC-J2 cells against ZEN-induced cell death and oxidative stress. We detected the intracellular level of ROS, MDA, T-AOC and the intracellular activity of SOD, CAT, GPx, TrxR. To clarify the potential molecular mechanisms, we investigated the effect of DL-SeMet on antioxidant enzyme: SOD, CAT, GPx, HO-1, TrxR1 and Phase II detoxification enzyme: NQO1, GST. We also detected signaling molecules of Nrf2 and NQO1 by Western blot analysis using specific antibodies.

## 2. Results

### 2.1. Experiment on Cell Viability

**Figure 1:**
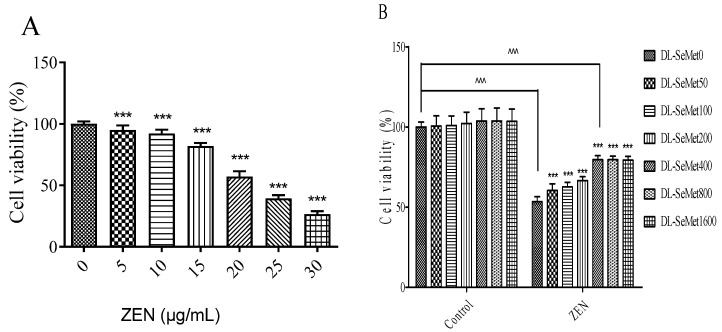
Effects of ZEN (**A**) on cell viability and protective effects of DL-SeMet (**B**). (**A**) The cells were exposed to ZEN (0, 5, 10, 15, 20, 25, and 30 μg/mL) for 24 h. (**B**) The cells were incubated with ZEN (20 μg/mL) and/or DL-SeMet (0, 50, 100, 200, 400, 800, 1600 ng/mL) for 24 h. Note: Cellular viability was determined by the MTT test. The results are expressed as a percentage of the control. Three independent experiments were performed, and the average data were shown. Bars represent mean ± SD of the three measurements. ^, ^^, and ^^^ means significantly different with controls at *p* < 0.05, *p* < 0.01, and *p* < 0.001, respectively. *, **, and *** means significantly different with controls at *p* < 0.05, *p* < 0.01, and *p* < 0.001, respectively.

The cell viability results are shown in Figure 1. MTT assays were used to determine the cell viability. As shown in Figure 1A, the results of cell viability observed a dose-dependent manner; the ZEN concentration at 30 μg/mL showed significant effects. The IC50 values were 22.15 ± 0.3 μg/mL. After that, ZEN (20 μg/mL) was chosen for these further experiments.

The protective effects of DL-SeMet on cell viability were investigated in the present study. As shown in Figure 1B, DL-SeMet did not have cytotoxicity towards cells. When DL-SeMet (400, 800, 1600 ng/mL) was co-administered with ZEN (20 μg/mL) for 24 h, the cell death induced by ZEN was obviously inhibited. However, the highest DL-SeMet concentration (1600 ng/mL) showed no better protective effects that compared with 400 ng/mL.

### 2.2. DL-SeMet Alleviates ZEN-Induced ROS Production

**Figure 2:**
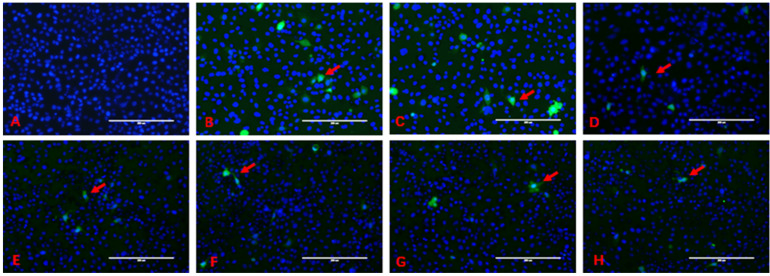
The intracellular production of ROS. (**A**), control group; (**B**), cells exposed with 20 μg/mL of ZEN; (**C**–**H**), ZEN + DL-SeMet (50, 100, 200, 400, 800, 1600 ng/mL). Cells were treated for 24 h. The arrow indicates abnormally shaped cells. Scale of bar is 200 μm.

The intracellular production of ROS is shown in Figure 2. The blue fluorescence is the nucleus stained by Hoechst 33342, and ROS are stained by DCFH-DA with the green fluorescence; ROS are located in the cytoplasm. Green fluorescence envelops blue fluorescence. In the control group, large quantities of cells had uniform distribution in the field of vision, and the green fluorescence in the cells was not obvious.

In the ZEN treatment group, the intercellular space was large than control and the number of cells was less than control, and more green fluorescent cells could be seen in the visual field. The green fluorescence intensity in the ZEN+DL-SeMet treatment group decreased gradually with the increase of DL-SeMet concentration, which was significantly different from that in the ZEN treatment group.

### 2.3. DL-SeMet Alleviates ZEN-Induced ROS Production

**Figure 3:**
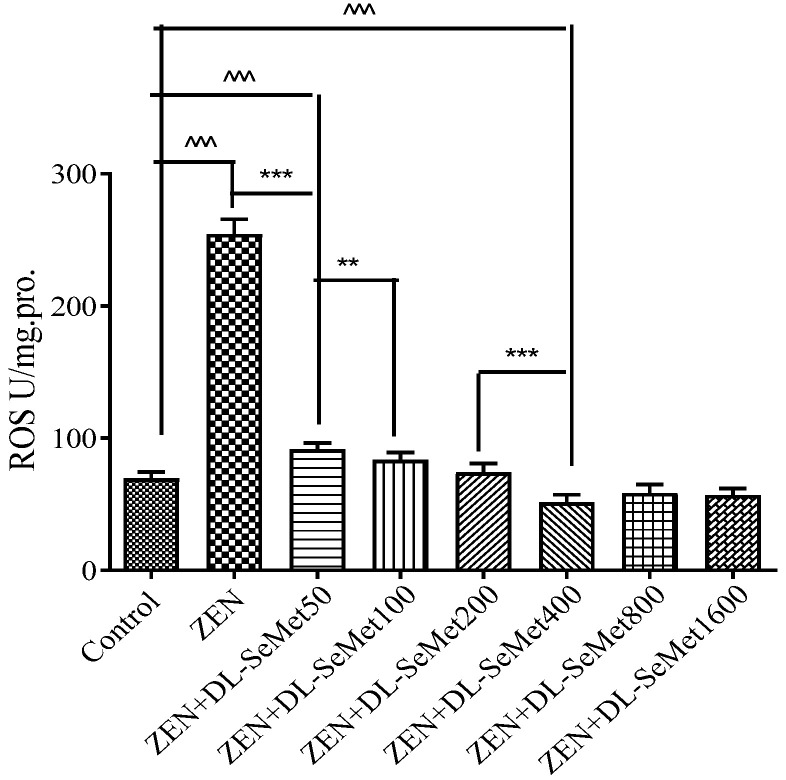
The level of intracellular ROS production. The cells were incubated with ZEN (20 μg/mL) and/or DL-SeMet (50, 100, 200, 400, 800, 1600 ng/mL) for 24 h. All the other notes were the same as in Figure 1.

The content of ROS production results is shown in Figure 3. For the oxidative damage changes’ determination, IPEC-J2 cells were exposed to ZEN (20 μg/mL) with different concentrations of DL-SeMet (50, 100, 200, 400, 800, 1600 ng/mL). As a result of oxidizing species concentration, ZEN-exposed cells increased compared with the basal rate. At all test concentrations, the reactive oxygen species production was observed to be concentration-dependent. Intracellular ROS production significantly decreased in ZEN+DL-SeMet 50 (*p* < 0.05). The highest intensity of fluorescence in IPEC-J2 cells was observed at ZEN, and the lowest intensity of fluorescence in IPEC-J2 cells was observed at ZEN+DL-SeMet 400 compared to controls.

### 2.4. DL-SeMet Attenuates ZEN-Stimulated Lipid Peroxidation

**Figure 4:**
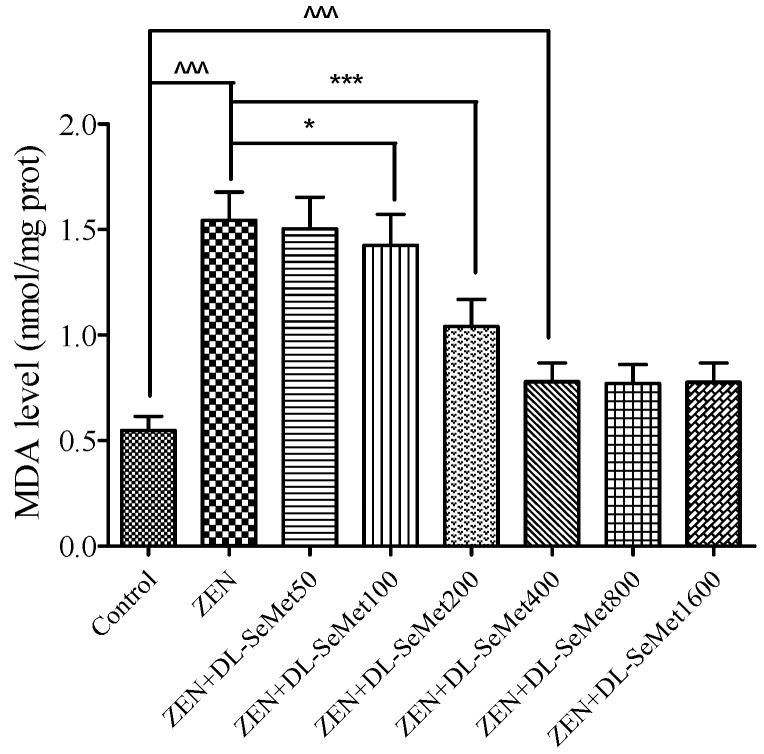
The effects of different concentrations of ZEN and the protective role of DL-SeMet on MDA production in IPEC-J2 cells. The cells were incubated with ZEN (20 μg/mL) and/or DL-SeMet (0, 50, 100, 200, 400, 800, 1600 ng/mL) for 24 h. All the other notes were the same as in Figure 1.

The MDA production results are shown in Figure 4. It was observed that the MDA level in ZEN treatment was higher (*p* < 0.05) than those in the control treatment; moreover, the highest level was observed in the ZEN treatment (1.54 ± 0.13 nmol/mg protein). The treatment of all doses of DL-SeMet significantly decreased the cell MDA levels.

### 2.5. DL-SeMet Prevents ZEN-Induced Decrease in Enzymatic Antioxidants

**Figure 5:**
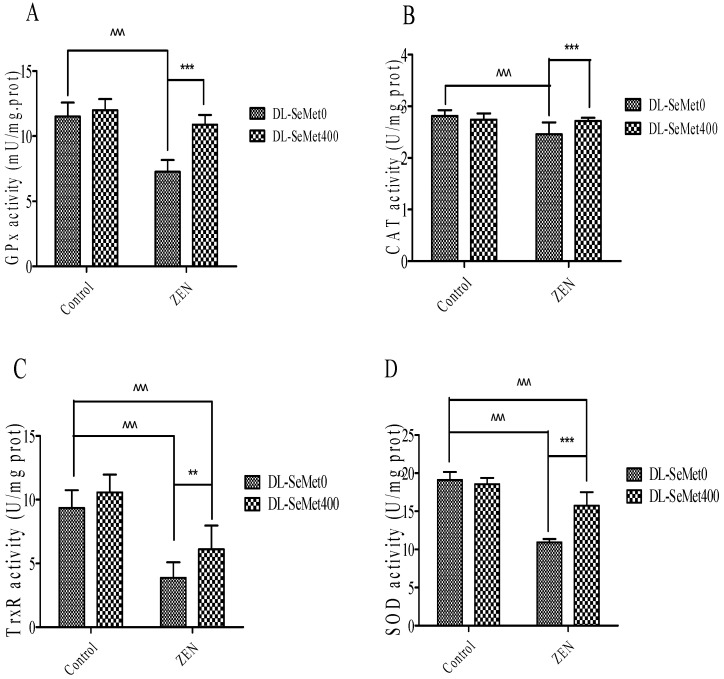
The effects of different concentrations of ZEN and the protective role of DL-SeMet on activities of enzymatic antioxidants (**A**, GPx activity; **B**, CAT activity; **C**, TrxR activity; **D**, SOD activity) in IPEC-J2 cells. The cells were incubated with ZEN (20 μg/mL) and/or DL-SeMet (400 ng/mL) for 24 h. All the other notes were the same as in Figure 1.

The enzymatic antioxidants’ activity results are shown in Figure 5. This illustrates the activities of enzymatic antioxidants, namely GPx, CAT TrxR1, SOD and in the IPEC-J2 cells. A significant (*p* < 0.05) decrease of the enzymatic antioxidant activities in ZEN-treated IPEC-J2 cells was observed. ZEN treatment with the addition of DL-SeMet increased (*p* < 0.05) the activities of enzymatic antioxidants in the cells.

### 2.6. DL-SeMet Normalizes the mRNA Expression of Nrf2, Keap1 and Enzymatic Antioxidants in ZEN Treated IPEC-J2 Cells

**Figure 6:**
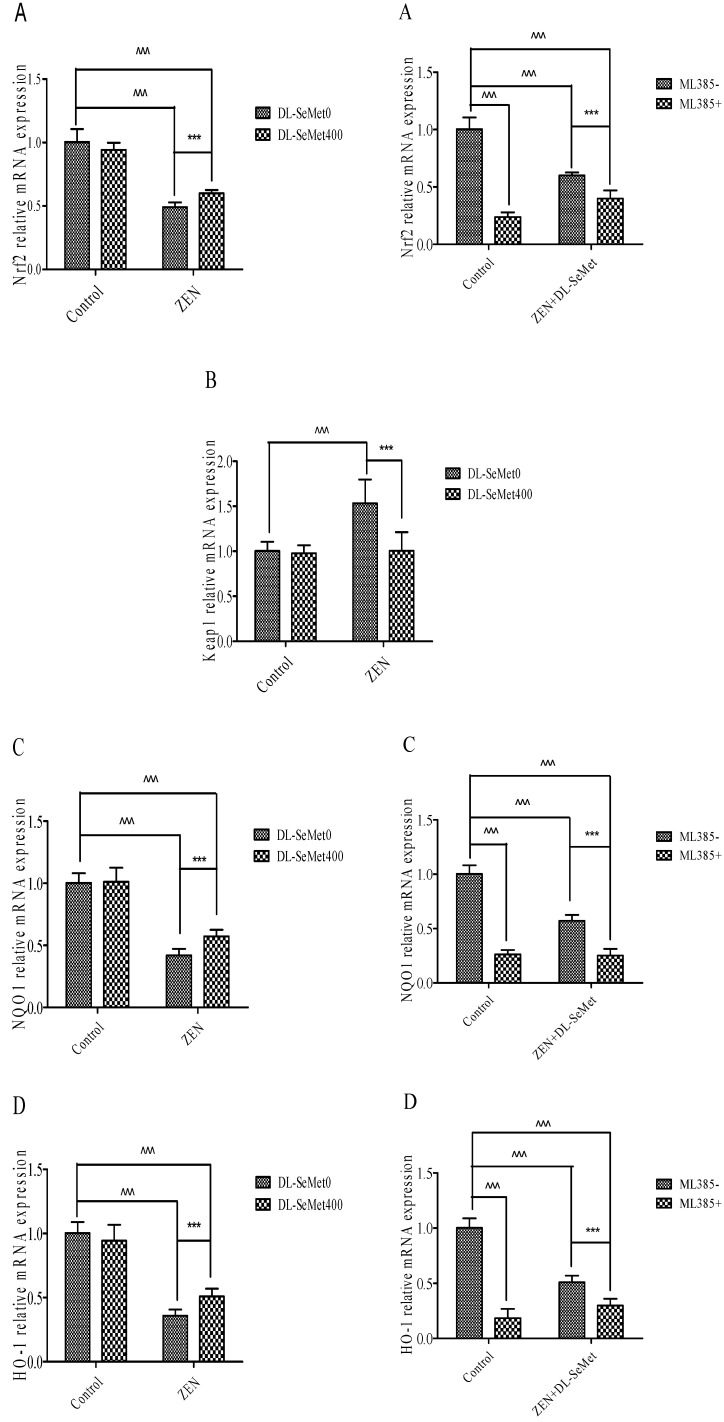

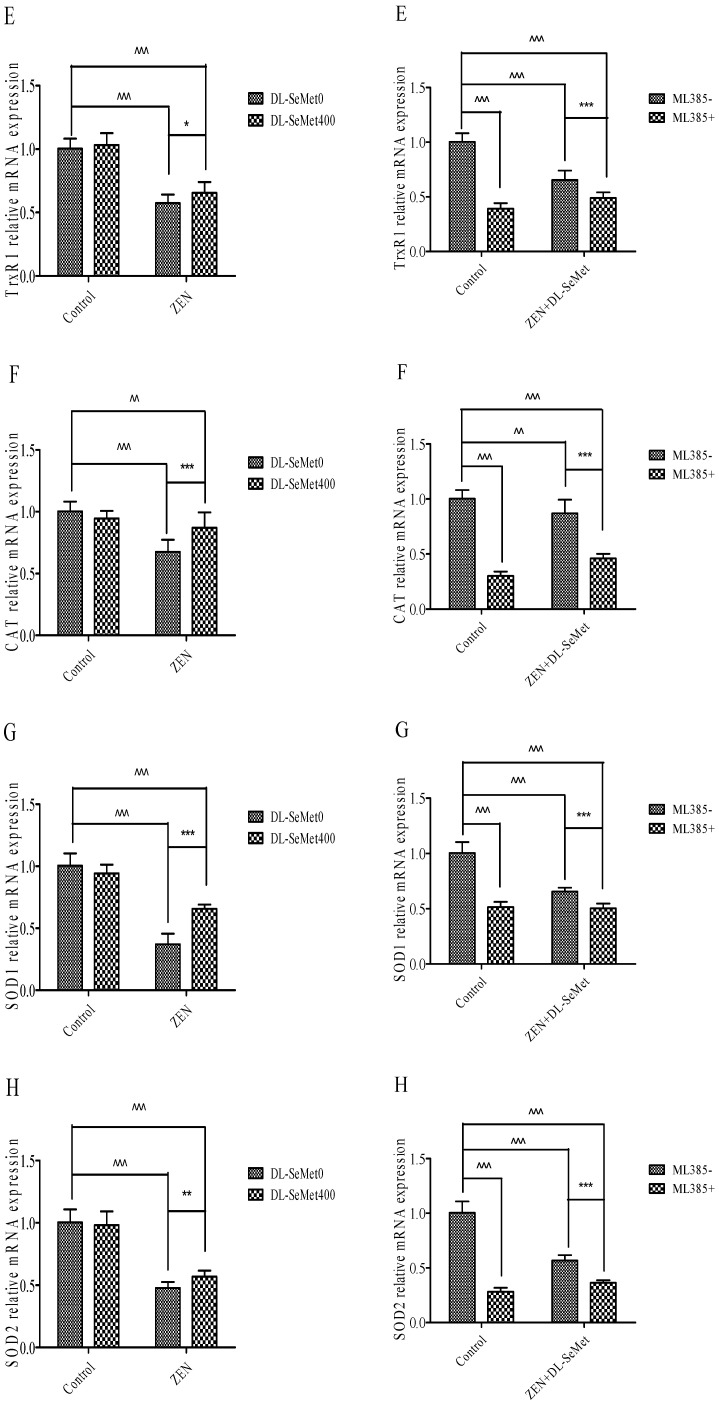

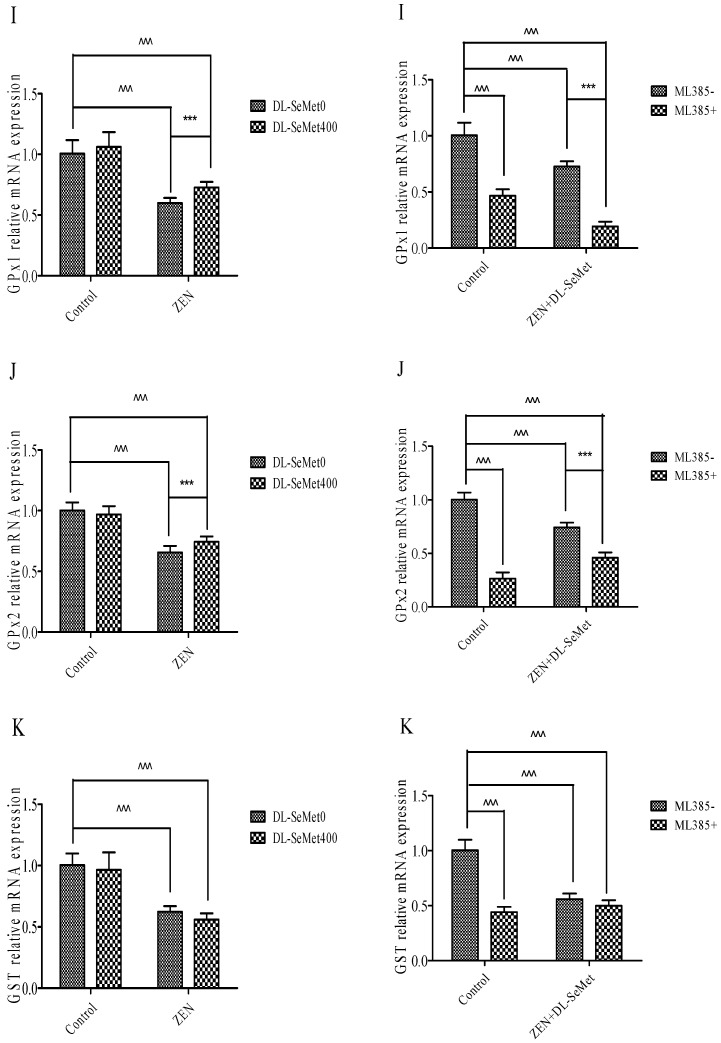
Individual effects of ZEN and the protective role of DL-SeMet on oxidative-related mRNA expression of IPEC-J2 cells (**A**, Nrf2 mRNA expression; **B**, Keap1 mRNA expression; **C**, NQO1 mRNA expression; **D**, HO-1 mRNA expression; **E**, TrxR1 mRNA expression; **F**, CAT mRNA expression; **G**, SOD-1 mRNA expression; **H**, SOD-2 mRNA expression; **I**, GPx1 mRNA expression; **J**, GPx2 mRNA expression; **K**, GST mRNA expression). Cells were exposed to 20 μg/mL ZEN and/or combinations of 400 ng/mL DL-SeMet for 24 h. All the other notes were the same as in Figure 1.

The results of mRNA expression are depicted in Figure 6. In these ZEN-intoxicated cells, the expression of Nrf2 and its down regulatory genes declined (*p* < 0.05) and there was simultaneous elevation of the Keap1 gene, whereas these altered mRNA expressions were significantly (*p* < 0.05) normalized in the ZEN-treated cells administered with DL-SeMet. To the contrary, cells treated with DL-SeMet alone did not demonstrate any statistical difference in comparison to that of the control cells.

Nrf2 levels significantly decreased in 20 μg/mL ZEN (*p* < 0.05) (Figure 6A). In the ZEN-SeMet groups, the expression of Nrf2 was higher (*p* < 0.05) than that in the mycotoxin alone (Figure 6A). Treating with 400 ng/mL DL-SeMet upregulated the expression of Nrf2 in the ZEN treatments. However, Nrf2 expression of ML385+ZEN+DL-SeMet was lower (*p* < 0.05) than that of mixtures of ZEN+DL-SeMet.

On the contrary, the Nrf2 and Keap1 expressions in 20 μg/mL ZEN treatment were both higher (*p* < 0.05) than that in the control (Figure 6B). The expression of Keap1 in the ZEN+DL-SeMet mix was lower (*p* < 0.05) compared with the 20 μg/mL ZEN-treated group (Figure 6B). In the 400 ng/mL DL-SeMet group, the reduction of Keap1 expression induced by ZEN was restored (Figure 6B).

Compared with the control treatment, NQO1 mRNA levels decreased significantly (*p* < 0.05) in 20 μg/mL ZEN (Figure 6C). The highest expression of NQO1 was shown in DL-SeMet (1.27 ± 0.27) (Figure 6C). Treatment with 400 ng/mL DL-SeMet significantly upregulated the expression of NQO1 in ZEN+DL-SeMet treatments (*p* < 0.05). However, in the ML385+ZEN+DL-SeMet, NQO1 expression was significantly lower than in ZEN+DL-SeMet treatments (*p* < 0.05).

Similar to mRNA expression NQO1, treatments with ZEN alone induced a significant (*p* < 0.05) decreased in HO-1. (Figure 6D). While, treatment with 400 ng/mL DL-SeMet significantly upregulated the expression of HO-1 in ZEN+DL-SeMet treatments (*p* < 0.05). However, in the ML385+ZEN+DL-SeMet, HO-1 expression was lower (*p* < 0.05) than DON ZEN-SeMet treatments.

Treatments with ZEN alone induced a significant (*p* < 0.05) decrease in TrxR1 (Figure 6E), while treatment with 400 ng/mL DL-SeMet significantly upregulated the expression of TrxR1 in ZEN+DL-SeMet treatments (*p* < 0.05). However, in the ML385+ZEN+DL-SeMet, TrxR1 expression was significantly lower than ZEN+DL-SeMet treatments (*p* < 0.05).

Treatments with ZEN alone induced a significant decrease in CAT (*p* < 0.05) (Figure 6F), while treatment with 400 ng/mL DL-SeMet significantly upregulated the expression of CAT in ZEN+DL-SeMet treatments (*p* < 0.05). However, in the ML385+ZEN+DL-SeMet, CAT expression was significantly lower than ZEN+DL-SeMet treatments (*p* < 0.05).

Treatments with ZEN alone induced a significant decrease in SOD-1 (*p* < 0.05) (Figure 6G) while treatment with 400 ng/mL DL-SeMet significantly upregulated the expression of SOD-1 in ZEN+DL-SeMet treatments (*p* < 0.05). However, in the ML385+ ZEN+DL-SeMet, SOD-1 expression was significantly lower than ZEN+DL-SeMet treatments (*p* < 0.05).

Treatments with ZEN alone induced a significant decrease in SOD-2 (*p* < 0.05) (Figure 6H), while treatment with 400 ng/mL DL-SeMet significantly upregulated the expression of SOD-2 in ZEN+DL-SeMet treatments (*p* < 0.05). However, in the ML385+ZEN+DL-SeMet, SOD-2 expression was significantly lower than ZEN+DL-SeMet treatments (*p* < 0.05).

Treatments with ZEN alone induced a significant decrease in GPx1 (*p* < 0.05) (Figure 6I). However, treating with 400 ng/mL DL-SeMet significantly upregulated the expression of GPx1 in ZEN+DL-SeMet treatments (*p* < 0.05). However, in the ML385+ZEN+DL-SeMet, GPx1 expression was significantly lower than ZEN+DL-SeMet treatments (*p* < 0.05).

Treatments with ZEN alone induced a significant decrease in GPx2 (*p* < 0.05) (Figure 6J) while treatment with 400 ng/mL DL-SeMet significantly upregulated the expression of GPx2 in ZEN+DL-SeMet treatments (*p* < 0.05). However, in the ML385+ ZEN+DL-SeMet, GPx2 expression was significantly lower than ZEN+DL-SeMet treatments (*p* < 0.05).

Treatments with ZEN alone induced a significant decrease in upregulation in GST (*p* < 0.05) (Figure 6K), while treatment with 400 ng/mL DL-SeMet significantly upregulated the expression of GST in ZEN+DL-SeMet treatments (*p* < 0.05).

### 2.7. DL-SeMet Regularizes the Protein Expression of Nrf2 and NQO1 in ZEN Treated IPEC-J2 Cells

**Figure 7:**
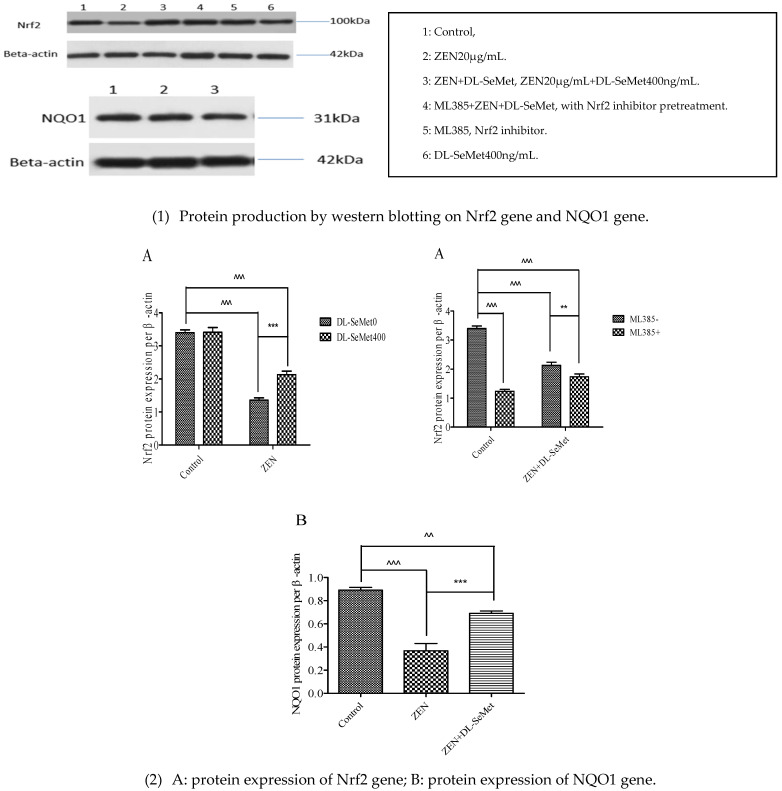
Individual effects of ZEN and the protective role of DL-SeMet on protein expression of Nrf2 and NQO1 in IPEC-J2 cells. ((**1**): Protein production by western blotting; (**2**): protein expression of Nrf2 and NQO1 genes) Cells were exposed to 20 μg/mL ZEN and/or combinations of 400 ng/mL DL-SeMet for 24 h. This graphical representation also includes the relative density of protein levels in IPEC-J2 cells, which were normalized to those of β-actin. All the other notes were the same as in Figure 1.

Regarding the effect of ZEN toxins and DL-SeMet on the expression of Nrf2 and NQO1 by Western blot analysis using specific antibodies, compared to control, no treatments have significantly changed (Figure 7).

## 3. Discussion

Zearalenone (ZEN), a Fusarium mycotoxin, is extremely toxic to rapidly dividing cells. ZEN not only causes significant reproductive toxicity, but also is a serious threat to the alimentary system, particularly for swine. It has been reported that ZEN induces cytotoxic and oxidative stress to PK15 cells and SIEC02 cells [7,8]. After 24 h of 5 μg/mL ZEN treatment, a significant decrease in cell viability was observed. Moreover, for ZEN the IC50 values by the MTT assay were 2.15 ± 0.3 μg/mL. The results of this experiment suggested that the cytotoxic effects of ZEN on IPEC-J2 cells were dose-dependent, which was in line with previous studies. For the determination of cell injury by ZEN, concentrations close to the IC50 of the ZEN (20 μg/mL) were chosen for subsequent experiments. Pig ovarian granulosa cells were treated with 60, 90, 120 μM ZEN. The results showed that the survival rate of porcine ovarian granulosa cells decreased significantly with the increase of ZEN concentration, and the content of ROS in cells increased significantly [15]. The toxicity of ZEN to RAW264.7 cells was studied at the concentration of 10 μM, 25 μM, and 50 μM. The results showed that ZEN could decrease the survival rate of RAW264.7 cells in a dose-dependent manner, increase the intracellular ROS, and eventually lead to cell death [16].

Selenium (Se) plays an important role in antioxidant defense, which is considered an essential micronutrient for humans and animals [17]. SeMet is an organic form of selenium-containing compounds widely found in plants and the main source of selenium in animals [18]. Studies have shown that the addition of 4 μM SeMet can improve the survival rate of pig alveolar macrophages (3D4/21) [19]. Researchers have discussed that selenium alleviates porcine nephrotoxicity of ochratoxin A by improving selenoenzyme expression in vitro [20]. Some studies have shown that sodium selenite has a protective effect on the oxidative stress injury of spleen lymphocytes induced by DON in piglets. Compared with the DON treatment, the cell survival rate and the activities of antioxidant enzymes SOD and CAT increased after adding sodium selenite. The level of T-AOC in cells increased and the content of MDA in cells decreased [21]. L-SeMet has an antioxidant protective effect on HepG2 treated with H2O2 at 100, 250, and 500 ng/mL [22]. Selenium improves the expression of GPx1 and SELS resistance to oxidative damage of primary pig spleen cells induced by AFB1 [23].

However, the reports on DL-SeMet alleviating the oxidative toxicities of ZEN on IPEC-J2 cells are limited. In the present study, it was shown that the anti-oxygenation effects of DL-SeMet against ZEN using the IPEC-J2 cell line and DL-SeMet itself had no significant effect on the survival rate and growth status of IPEC-J2 cells. In ZEN-treated IPEC-J2 cells, the ROS (reactive oxygen species) levels increased, which indicated that oxidative stress had occurred. In previous studies conducted in our laboratory, ZEN was seen to cause cytotoxic effects and oxidative stress in IPEC-J2 cells. Taking into account that ROS generation can be a cause or consequence of mitochondrial alterations, these mycotoxins can damage cell function and/or structure. Similarly, Tatay E. et al. (2016) suggested that ZEA (6.25–25 μM) and its metabolites affect the redox status, increasing ROS production in CHO-K1 cells [9]. A time-dependent increase in ROS production was also reported by Venkataramana et al. (2014) in human neuroblastoma cells (SH-SY5Y) exposed to ZEA (25–200 μM) over a period of 24 h [24]. Compared with the ZEN treatment group, the survival rate of cells was significantly improved by adding DL-SeMet. When the concentration of DL-SeMet was 400 ng/mL, 800 ng/mL, and 1600 ng/mL, the cell survival rate was higher, and the cell survival rate (%) increased from 53.36 ± 3.18 (ZEN group) to 79.60 ± 2.57 (DL-SeMet 400 ng/mL), respectively. Reactive oxygen species (ROS) act as intracellular signaling molecules in the regulation of receptor activator of nuclear factor-κB ligand (RANKL)-dependent osteoclast differentiation, but they also have cytotoxic effects that include peroxidation of lipids and oxidative damage to proteins and DNA.

The results of this experiment indicated that the DL-SeMet has the antioxidant ability against the oxidative stress caused by ZEN. Furthermore, there is positive proof that ROS are critically involved in the aberrant signaling and cell injury. In this experiment, it was shown that ZEN intoxication improved the expression of ROS production, which leads a variety of disorders, including an increase in the cell death ratio, lipid peroxidation, and reduction of the enzymatic antioxidants level. The addition of DL-SeMet significantly enhanced the expression of the enzymes, therefore relieving the ZEN-induced oxidative stress in the IPEC-J2 cells. Moreover, it was suggested that increased ROS production is associated with ZEN-mediated cytotoxicity and oxidative injury. DL-SeMet treatments were effective in reducing the generation of ROS. It was in line with the results of the current study that the treatment of DL-SeMet suppressed the lipid peroxidation and maintained the antioxidant defense mechanisms in the ZEN-exposed IPEC-J2 cells.

Cellular protective mechanisms against oxidative stress include transcriptional control of cytoprotective enzymes by the transcription factor, nuclear factor E2-related factor 2 (Nrf2). Antioxidant enzymes such as SOD, CAT, GPx, HO-1, NQO1, and TrxR1 are recognized as the primary intracellular enzymes that defend against oxidative stress. The addition of 1 μM SeMet did not increase the expression of GPx gene mRNA in human umbilical vein endothelial cells (HUVEC), but when the activity of GPx was inhibited, the addition of 1 μM SeMet could increase the GPx gene mRNA of human umbilical vein endothelial cells [25]. GPx as a selenoprotein, belonging to the first and second levels of antioxidant networks, is involved in maintaining redox balance and signal transduction. Over the years, a great deal of evidence has been accumulated to confirm the importance of the enzyme in eukaryotes [26]. The levels of these enzymes are pertinent indirectly to evaluate the antioxidant–pro-oxidant condition in ZEN toxicity. Lower concentrations of SOD activity in ZEN-treated cells reflect the augmented capacity of superoxide radical anions. The ZEN-augmented superoxide radicals also have the ability to inhibit the action of CAT. Furthermore, in order to protect against ROS-mediated oxidative insult, the glutathione dependent enzymes such as GPx, GST, and GR are the essential antioxidant enzymes. Significant decreases were observed in the activities of these enzymes in the ZEN-intoxicated cells. This might be due to the interaction of these –SH groups with the lipid peroxidation products. The inactivation exacerbated the cells into heavier oxidative damage. However, DL-SeMet treatment may markedly renew the damage of the antioxidant defense system, perhaps because of its strong antioxidant and free-radical scavenging. The results showed that ZEN significantly decreased the level of T-AOC and the activities of SOD, CAT, GPx, and TrxR. Compared with the ZEN group, the intracellular T-AOC level and SOD, CAT, GPx and TrxR activity in ZEN DL-SeMet group increased significantly. These results suggest that DL-SeMet may play a protective role against oxidative stress by increasing the activity of antioxidant enzymes. This is consistent with the results of previous reports.

The Nrf2/Keap1-ARE signaling pathway can resist oxidative stress induced by internal and external substances or conditions [27]. After recognition and a combination of Nrf2 and ARE, the downstream II metabolic enzymes (NQO1, GST) and antioxidant enzymes (SOD, CAT, GPx, TrxR, HO-1, etc.) can be regulated, and antioxidant stress can be played in the animal body [28]. Compounds carrying this reactive group have been reported to induce HO-1 expression through the activation of Nrf2 nuclear translocation [29]. It is reported that selenium can antagonize the hepatotoxicity of arsenic by activating the Nrf2 signaling pathway [14]. Free Nrf2 translocates to the nucleus, regulating transcriptionally and then activating the genes encoding phase II detoxification or antioxidant enzymes. DL-SeMet promotes the heme oxygenase-1 expression, which leads to the activation of the Nrf2 gene. The antioxidant ARE regulated the genes encoding detoxification enzymes and antioxidant proteins in a transcriptional way, which plays an important role in the cellular defense system. Furthermore, the inactivation of Nrf2 was reported in the cytoplasm with Keap1. ARE activation causes the signals from the protein kinase pathways, disrupting the Nrf2-Keap1 complex and leading to nuclear translocation of Nrf2. After the activation, Nrf2 binds to ARE sites in the promoter regions of many and antioxidant genes, leading to the coordinate upregulation of downstream targets that enhance the cellular detoxification processes and antioxidant potential.

The results showed that ZEN significantly downregulated the mRNA expression of Nrf2, Keap1, SOD1, SOD2, GPx1, GPx2, TrxR1, CAT, HO-1, NQO1, and GST genes and upregulated the mRNA expression of the Keap1 gene. This is consistent with the results of oxidative stress damage and decrease in antioxidant capacity induced by ZEN in this experiment. Compared with the ZEN group, the ZEN+DL-SeMet group significantly upregulated the expression of the Nrf2, Keap1, SOD1, SOD2, GPx1, GPx2, TrxR1, CAT, HO-1, and NQO1 genes and downregulated the mRNA expression of the Keap1 gene. This is consistent with the results of DL-SeMet reducing oxidative stress damage induced by ZEN in IPEC-J2 cells. The expression of the GST gene had no change, which could be due to the fact that GST is a kind of selenium-free GPx, and DL-SeMet is the donor of selenium, which plays an antioxidant role through selenium [30]. Therefore, the mRNA expression of the GST gene was not observed by adding DL-SeMet in this experiment.

It was observed that ZEN intoxication downregulated the anti-oxidative Nrf2 and NQO1 proteins, which demonstrated the oxidative ability of ZEN. However, the administration of DL-SeMet effectively hampered all these ZEN-caused intrinsic pro- oxidative events.

The results showed that compared with the ZEN+DL-SeMet group, the addition of ML385 significantly decreased the mRNA expression of Nrf2 and the expression of Keap1, and the downstream target genes SOD1, SOD2, GPx1, GPx2, CAT, TrxR1, HO-1, and NQO1 were also downregulated. Compared with the ZEN+DL-SeMet group, the addition of ML385 significantly decreased the protein expression of Nrf2 and NQO1.

The outcomes of the present study indicate that the administration of DL-SeMet significantly attenuated the ZEN-induced oxidative toxicity in IPEC-J2 cells. The antioxidant activities might be regarded as the key aspects responsible for the cell-protective nature of DL-SeMet. Therefore, it considered a prospective therapeutic choice in preventing the oxidative intestinal injury and the dysfunction resulting from ZEN intoxication.

## 4. Conclusions

In conclusion, ZEN can induce oxidative stress by subsequent suppression of the expression of antioxidant stress pathway-related genes species, and these results suggested that the oxidative pathway plays an important role in ZEN toxins-induced cell oxidative stress and cell death. DL-SeMet effectively protects the IPEC-J2 cells in Nrf2/Keap1 pathways in ZEN-caused oxidative toxicity. The ameliorative effect in this study was mainly due to the inhibition of ROS formation, thereby inhibiting the peroxidation of membrane lipids and preventing cell death. In addition, DL-SeMet administration significantly improves the levels of enzymatic activity in IPEC-J2 cells, which further contributes to its cell-protective effect. Overall, the findings of this study suggested that antioxidants such as DL-SeMet are important to treat and prevent the toxicity of ZEN toxins.

## 5. Materials and Methods

### 5.1. Chemicals and Reagents

The reagent grade chemicals ZEN, DL-SeMet, [3-(4,5-dimethylthiazol-2-yl)-2,5- diphenyltetrazolium bromide] and dimethyl sulfoxide were obtained from Sigma-Aldrich (St. Louis, MO, USA). Dulbecco’s modified Eagle’s medium-F:12 cell culture medium was purchased from GE Healthcare Life Sciences (Beijing, China). Fetal bovine serum (FBS) was obtained from Gibco (Invitrogen Corporation, Grand Island, NY, USA). ML385 (MCE, Cat. No.: HY-100523, CAS No.: 846557-71-9) were commercially obtained. Phosphate-buffered saline and Micro Oxidized Thioredoxin Reductase (TrxR) Assay Kit were purchased from Solarbio Life Sciences (Beijing, China). Porcine reactive oxygen species ELISA Kit was supplied by Shanghai Enzyme-linked Biotechnology (Shanghai, China). Antibodies against Nrf2 (Abcam, ab92946), NQO1 (Sangong Biotech Co., Ltd., D261049) were purchased commercially. All other chemicals were obtained from Beyotime Institute of Biotechnology (Nantong, China).

Ethanol at a concentration of 0.17% was chosen as an organic solvent for the dilution of ZEN pure product for subsequent testing. ML385 was dissolved in DMSO and DL-SeMet was dissolved in water. The final concentration of the DMSO used as a solvent in the culture medium was 0.05%.

### 5.2. Cell Culture and Treatment

The Porcine Small Intestinal Epithelial Cell Line was donated by China Agricultural University.

The preparation of IPEC-J2 cells was according to the previous study from this lab [31]. In brief, IPEC-J2 cells were grown and passaged in DMEM-F:12 supplemented with 10% FCS and 1% Pen/Strep. The cultures were maintained in a humidified atmosphere of 95% air and 5% CO_2_ at 37 °C. Cells were passaged at pre-confluent densities by the use of a solution containing 0.05% trypsin and 0.5 mM EDTA (Beyotime Institute of Biotechnology, Nantong, China). The cells were seeded with a starting density of 1.0 × 10^5^ cells/25 cm^2^ cell culture flask (Nest, Eimer Biotechnology Company, Wuxi, China), 5000 cells/well in 96-well plates and 0.5 × 10^6^ cells/well of a 6-well plate. Subsequently, once the cells were confluent to approximately 80~90%, the cell monolayer was washed with PBS and the culture medium was replaced for subsequent testing. For all the experiments, IPEC-J2 cells cultured between the ninth and twentieth passages were used.

For ML385 supplement, cells were plated in complete culture media with 2 μg/mL for 1 h, then the medium was exchanged and supplemented with other treatment.

### 5.3. Measurement of Cell Viability

Cytotoxicity of ZEN was determined using the MTT method. Cells were treated with ZEN (0, 5, 10, 15, 20, 25, and 30 μg/mL) in serum-free media for 24 h. At the end of treatments, the cell monolayer was incubated in 0.5 mg/mL MTT solution and incubated at 37 °C for 3 h; in the viable cells, purple formazan would have formed. Then, DMSO was used to solubilize and they were shaken for 10 min. The cell viability was quantified by measuring the absorbance of purple formazan at a wavelength 570 nm on a microplate reader (TECAN Austria GmbH, Salzburg, Austria) and expressed as the relative formazan formation in treated samples as compared to control cells ((A570 treated cells/A570 control cells) 100%) after correction for background absorbance [32]. Estimated the IC50 values of IPEC-J2 cells viability figure by Graph Pad Prism 5.0 Software. Then the IC50 concentrations of ZEN induced to IPEC-J2 cells were referenced by subsequent study.

In addition, to assess the protective role of DL-SeMet in ZEN-induced cytotoxicity, DL-SeMet (0, 50, 100, 200, 400, 800, 1600 ng/mL) was co-administered with ZEN for 24 h. The experiments were repeated three times with five replicates for each treatment

### 5.4. Determination of Intracellular ROS

Intracellular ROS production was monitored in IPEC-2 cells by adding 2′, 7′-Dichlorofluorescin diacetate (DCHF-DA). Briefly, IPEC-2 cells were seeded in 12-well plates and incubated for 24 h at the density of 0.5×106 cells/well. After treatment, the treated cells were rinsed three times using PBS and incubated with DCFH-DA (10 μM) and Hoechst 33342 (5 μg/mL) in serum-free DME/M F12 medium at 37 °C and 5% CO_2_ for 25 min according to the manufacturer’s instructions. Lastly, we observed the ROS in cells by scanning with a fluorescence microscope (Olympus Corporation, Tokyo, Japan). Intracellular ROS were marked by green fluorescent light in the microscope. The nuclear chromatin was marked by blue fluorescent light in the microscope.

### 5.5. Evaluation of ROS Level

Cells were treated as described for MDA assay. Intracellular ROS production was monitored using the ELISA method, ROS content was assessed using a porcine reactive oxygen species ELISA Kit according to the manufacturer’s instructions. The results were expressed as U per mg protein.

### 5.6. Lipid Peroxidation (MDA) Assay

Cells were seeded in 6-well plates and incubated for 24 h. After treatment, the cells were rinsed three times using PBS and incubated with ZEN (0, 5, 10, 15, 20, 25, and 30 μg/mL) in serum-free media for 24 h. Similarly, the cells were treated with DL-SeMet (0, 50, 100, 200, 400, 800, and 1600 μg/mL). After all treatments were completed, cells were washed twice with PBS, then lysed and collected. The level of lipid peroxidation was measured via the 2-thiobarbituric acid color reaction for malondialdehyde by using the kit according to the manufacturer’s instructions. The results were expressed as nmol per mg protein.

### 5.7. Antioxidant Enzymes Activity Assays

Cells were treated as described for MDA assay. Protein concentration and the activity of Thioredoxin Reductase, Total Antioxidant Capacity, Glutathione peroxidase (containing selenium), total Glutathione peroxidase, total superoxide dismutase and Catalase activity were assessed using kits (kit number: S0101, S0051, S0116, and S0053, Beyotime Ltd., Shanghai, China) according to the manufacturer’s instructions. The results were expressed as U per mg protein.

### 5.8. Quantitative Real-Time PCR

Total RNA was extracted from IPEC-J2 cell samples by the TRIzol kit (Invitrogen) according to the manufacture’s protocol. The real-time PCR analyses of the samples were conducted as described by Zhao et al. [33]. As shown in Table 1, the primers were designed from published GenBank sequences and were synthesized by Sangon (Shanghai, China).

### 5.9. Western Blot Analysis

At the end of indicated treatment, preparation of total cellular proteins. The Western blot analyses of these samples were performed as previously [34]. After blocking with 5% BSA in fresh TBS buffer containing 0.05% Tween-20 at 37 °C for 2 h, the membranes were probed with the first antibody including rabbit anti-Nrf2, anti-NQO1, anti-β-actin antibody, at 4 °C overnight following 1 h incubation in corresponding secondary antibodies (dilution, 1: 5000) at room temperature. The blot in membranes was visualized by an enhanced chemiluminescence (ECL) Plus detection system (P1010, Applygen, Beijing, China). The blots were quantified by Quantity One software (Bio-Rad, Hercules, CA, USA). The concentration value of each cellular protein expression was expressed as each normalized data relative to control.

### 5.10. Statistical Analysis

Each experiment was done separately. Data were preliminarily calculated by using Microsoft Excel 2016 (Microsoft Corporation, Redmond, WA, USA). Statistical differences were conducted using Statistical Product and Service Solutions Software (SPSS Inc., Chicago, IL, USA). The significance was considered to be at the probability level of *p* < 0.05. IC50 and was analyzed by Graph Pad Prism 5.0 (Graph Pad Software, Inc., San Diego, CA, USA).

## Figures and Tables

**Table 1:** List of primer sequences for qPCR.

Genes	Sequences (5′ → 3′)	Fragments Size (bp)	Accession Number
GAPDH	FP: GGAAGCTGTGGCGTGATGGC RP: TTCTCCAGGCGGCAGGTCAG	175	NM-001206359.1
Nrf2	FP: CCAATTCAGCCAGCACAACACATC RP: GACTGAGCCTGGTTAGGAGCAATG	149	XM_021075133.1
Keap1	FP: GGAGGACCACACCAAGCAAGC RP: GGATGAAGCCAGCACCACCTTG	142	NM-001114671.1
CAT	FP: ACGCCTGTGTGAGAACATTG RP: GTCCAGAAGAGCCTGAATGC	124	NM_214301.2
HO-1	FP: AGGCTGAGAATGCCGAGTTC RP: TGTGGTACAAGGACGCCATC	90	NM_001004027.1
NQO1	FP: AGTATCCTGCCGAGACTGCTCTG RP: CACAAGGTCTGCGGCTTCCAC	95	NM-001159613.1
TrxR1	FP: GCTCAAGTGCGGACTGACCAAG RP: AGCAACCGGCCTGGAGGATG	128	NM_214154.3
SOD1	FP: GCGAGTCATGGCGACGAAGG RP: GACCTGCACTGGTACAGCCTTG	191	NM_001190422.1
SOD2	FP: TGTATCCGTCGGCGTCCAAGG RP: TCCTGGTTAGAACAAGCGGCAATC	93	NM_214127.2
GPX1	FP: GCGTCGCTCTGAGGCACAAC RP: GGTCGGACGTACTTGAGGCAATTC	167	NM_214201.1
GPX2	FP: ATTCTTCCTGGCTCCTCCTTCCTC RP: AGGCTGATAGCACTGAGGTCGTAG	136	NM-001115136.1
GST	FP: GCCGAGGCAGAATGGAGTGTATC RP: GGTGGCGATGTAGTTGAGGATGG	197	NM-214389.2
GSH-Px	FP: CGTGTAACCAGTTCGGACATCAGG RP: CGCCATTCACCTCACACTTCTCG	129	AJ010340.1

FP forward primer, RP reverse primer.

## Data Availability

Not applicable.

## Abbreviations

ZEN, zearalenone; ROS, reactive oxygen species; SeMet, selenomethionine; IPEC-J2, porcine small intestinal epithelial cell line; MDA, malondialdehyde; T-AOC, total antioxidant capacity; SOD, superoxide dismutase; CAT, catalase; GPx, glutathione peroxidase; TrxR, thioredoxin reductase; HO-1, hemeoxygenase-1; NQO1, NAD(P)H quinone dehydrogenase 1; DMSO, dimethyl sulfoxide; DMEM, Dulbecco’s modified Eagle’s medium; FBS, fetal bovine serum; MTT, 3-(4,5-dimethylthiazol-2-yl)-2,5- diphenyltetrazolium bromide; IC50, half maximal inhibitory concentration; DCHF-DA, 2′, 7′-dichlorofluorescin diacetate; T-GSH-Px, total glutathione peroxidase; PVDF, polyvinylidene difluoride; ECL, chemiluminescence; PBS, phosphate-buffered saline; TBA, 2-thiobarbituric acid; PMSF, protease inhibitors.
